# General Anesthesia for Patients With Cyclic Vomiting Syndrome and Obesity: A Case Report

**DOI:** 10.7759/cureus.65130

**Published:** 2024-07-22

**Authors:** Hidetaka Kuroda, Rumi Kaneko, Norika Katagiri, Keita Kagawa, Natsuki Saito, Yoko Sasaki, Kazumi Kuroda-Ohgi, Yukiko Kuroda, Shinsuke Kuroda, Shota Tsukimoto, Noboru Ishikawa, Takahiro Abe, Takuro Sanuki

**Affiliations:** 1 Department of Dental Anesthesiology, Kanagawa Dental University, Kanagawa, JPN; 2 Department of Anesthesiology, Dokkyo Medical University Saitama Medical Center, Saitama, JPN; 3 Department of Dental Anesthesiology, Tokyo Dental College, Tokyo, JPN; 4 Department of Anesthesiology, Saitama Medical University International Medical Center, Saitama, JPN; 5 Prosthodontics, White Dental Clinic, Gunma, JPN; 6 Pediatric Dentistry, White Dental Clinic, Gunma, JPN; 7 Endodontics, White Dental Clinic, Gunma, JPN; 8 Department of Forensic Odontology and Anthropology, Tokyo Dental College, Tokyo, JPN; 9 Department of Oral and Maxillofacial Surgery, Kanagawa Dental University, Kanagawa, JPN

**Keywords:** hypothalamic-pituitary-adrenal axis, cortisol, anti-diuretic hormone, adrenocorticotropic hormone, day surgery, dental practice, general anesthesia, obesity, cyclic vomiting syndrome, intellectual disability

## Abstract

Cyclic vomiting syndrome (CVS) is a chronic digestive disorder characterized by recurrent episodes of severe nausea and vomiting. The perioperative management of patients with CVS undergoing general anesthesia is challenging, especially when combined with obesity. This case report describes the successful management of a patient with CVS and obesity who underwent dental surgery under general anesthesia.

A 21-year-old woman with CVS, obesity (body mass index, 35), and intellectual disability was scheduled for tooth extraction and composite resin restoration under general anesthesia. The patient was diagnosed with CVS at the age of 20 years with frequent vomiting attacks requiring hospitalization. Surgery was scheduled during the CVS remission to reduce the risk of perioperative vomiting. Preoperative laboratory test results were normal, including serum adrenocorticotropic hormone (ACTH), anti-diuretic hormone (ADH), and cortisol levels. General anesthesia was induced using remifentanil and propofol. Nasal endotracheal intubation was performed after rocuronium administration. Local anesthesia (2% lidocaine with 1:80,000 epinephrine) was used for all dental procedures. Postoperatively, midazolam was administered to control agitation. No postoperative vomiting occurred. Serum ACTH, ADH, and cortisol levels showed no significant changes before and after anesthesia, suggesting that hypothalamic-pituitary-adrenal (HPA) axis activation due to surgical stress did not occur.

This case highlights the importance of careful perioperative planning and monitoring stress-related hormone levels in patients with CVS or obesity. An anesthetic approach using midazolam may effectively suppress HPA axis activation and prevent postoperative vomiting.

## Introduction

Cyclic vomiting syndrome (CVS) is a chronic digestive disorder characterized by recurrent episodes of severe nausea and vomiting [1-3]. These episodes, which can occur with alarming frequency - up to several times per hour - may persist for a few hours to several days, significantly impacting the quality of life of affected individuals [3,4]. The etiology of CVS remains largely enigmatic, although various triggers have been identified, including psychological stress, certain foods, menstrual cycles, and infections [5,6]. While CVS is predominantly observed in pediatric populations, it is increasingly recognized as a condition that can affect individuals across all age groups [1-3]. The pathophysiology of CVS is multifaceted and not fully elucidated while current research suggests a complex interplay between the central nervous system, autonomic nervous system, and gastrointestinal function [1-3].

Obesity also increases the risk of vomiting via several mechanisms. Primarily, obesity leads to elevated intra-abdominal pressure and dysfunction of the lower esophageal sphincter, compromising the natural barrier that prevents retrograde flow of stomach contents into the esophagus [7-9]. Furthermore, obesity is associated with heightened gastric acid secretion; this excess acid can irritate both the stomach lining and the esophagus, potentially serving as a trigger for vomiting episodes [7-9].

Beyond the immediate discomfort and distress it causes, perioperative vomiting poses serious risks, including aspiration pneumonia and potential choking [9]. Despite the critical nature of this issue, there is a notable paucity of literature addressing the perioperative management of CVS under general anesthesia [10]. This gap in clinical guidance leaves healthcare providers without standardized protocols or specific recommendations for appropriate management of general anesthesia for CVS. This case report aims to address this gap by presenting a case of day surgery under general anesthesia in a patient with both CVS and obesity and seeks to contribute to the development of potentially appropriate management strategies for CVS in perioperative settings.

## Case presentation

**Figure 1:**
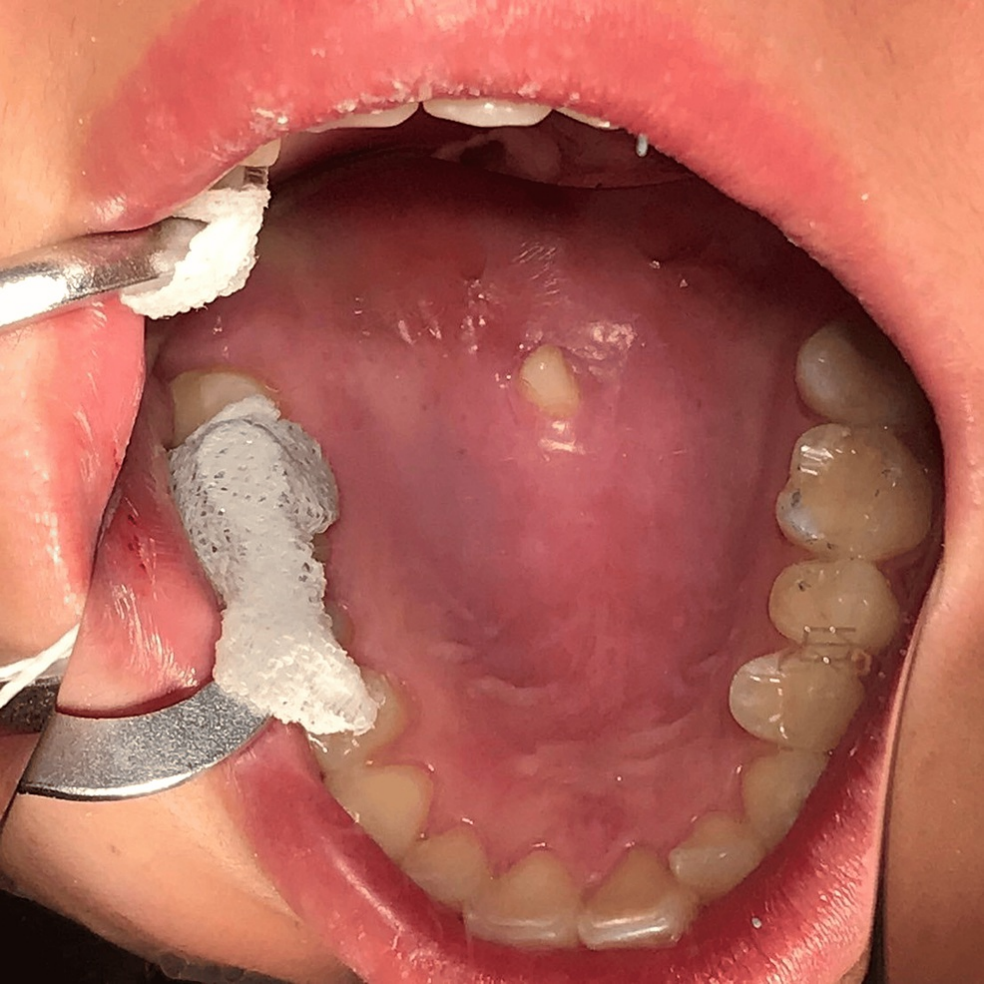
Intraoral photograph The image was captured using a mirror. The supernumerary tooth crown erupts in the middle of the palate.

**Figure 2:**
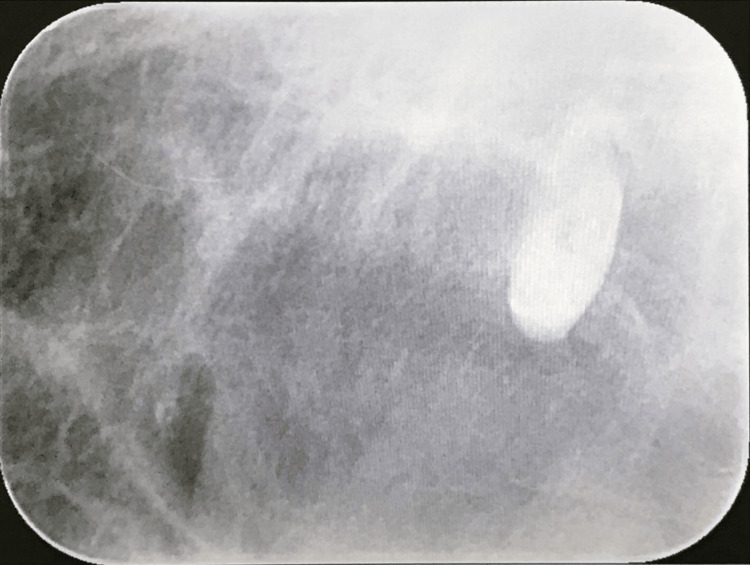
Intraoral radiograph The intraoral radiograph was taken using the occlusal radiographic technique.

A 21-year-old woman, who had an intellectual disability, was scheduled for the extraction of the maxillary central supernumerary tooth and composite resin restoration spanning several teeth under general anesthesia, as she was uncooperative in treatment. An intraoral photograph and occlusal radiograph of the maxillary central supernumerary are shown in Figures 1-2.

The patient had a history of epilepsy, obesity, and CVS. The epilepsy was controlled using oral sodium valproate. Her height and weight were 159 cm and 88 kg, respectively; her body mass index was 34.8 kg/m^2^. The Mallampati classification was class 3; however, other physical findings related to airway security could not be evaluated because of the contracting intellectual disabilities. CVS was diagnosed at the age of 20, and the patient had been in and out of the hospital due to frequent vomiting attacks. In addition to antiepileptic drugs, the patient was taking ramelteon, clomipramine, and risperidone. To reduce the risk of perioperative vomiting, surgery was scheduled outside the CVS seizure period. Preoperative biological laboratory tests revealed no abnormal findings, including blood levels of electrolytes, a serum adrenocorticotropic hormone (ACTH) level of 17.4 pg/mL (normal: 7.2-63.3 pg/mL), anti-diuretic hormone (ADH, also known as vasopressin) level of 0.6 pg/mL (normal: < 2.8 pg/mL for free drinking), and cortisol level of 5.98 µg/dL (normal: 4.5-21.1 µg/mL).

The patient was instructed to fast from midnight the day before and to abstain from clear liquid three hours before the induction of general anesthesia. Rapid induction of general anesthesia was performed in the semi-Fowler position on a dental chair, and remifentanil and propofol were administered. After falling asleep, facemask ventilation was achievable using an oral airway. After administering rocuronium to achieve muscle relaxation, nasal endotracheal intubation was performed using a video laryngoscope.

Before each surgery and dental treatment, infiltration anesthesia was performed using a total of 5.4 mL of 2% lidocaine hydrochloride with 1:80,000 epinephrine. A total of eight teeth required dental treatment. Seven teeth underwent composite resin restoration, and one tooth received an inlay restoration. Tooth extraction was possible using standard techniques. All procedures were completed without any problems.

After the administration of sugammadex, extubation was performed while the patient was awake, without any incidents. To control postoperative agitation, 4 mg of midazolam was administered by titration. We initially administered 3 mg of midazolam and then administered an additional 1 mg while evaluating the patient's condition, resulting in moderate sedation. The surgery lasted for 1 h 30 min, with a total anesthesia time of 2 h 55 min. Blood loss was minimal, and the total fluid administration was 400 mL.

The patient was observed for two hours after the general anesthesia. No postoperative vomiting was observed. After confirming that the patient could drink water and urinate on their own, she was allowed to go home. The serum ACTH levels measured before and after general anesthesia were 9.3 pg/mL and 7.8 pg/mL, ADH levels were 1.6 pg/mL and 1.2 pg/mL, and cortisol levels were 5.1 µg/dL and 4.0 µg/dL, respectively. The inlay was fitted under sedation two weeks later.

## Discussion

This case report describes the management of general anesthesia in a patient with CVS and obesity and offers insights into some of the complexities and challenges that can be involved in perioperative care for patients with these conditions.

CVS is a perplexing disorder characterized by sudden, severe, and recurrent episodes of nausea and vomiting interspersed with symptom-free intervals [1-6]. Known by various names, such as periodic ACTH-ADH release syndrome, acetonemic emesis, or autointoxication, CVS is classified as a migraine-related periodic syndrome according to the International Classification of Headache Disorders, 3rd edition [1,11]. The etiology of CVS is multifactorial, with various triggers identified, including mental and physical stress, infection, fatigue, dietary factors, and menstruation [5]. Among these, stress is considered the most common precipitant, highlighting the critical role of stress management in CVS care [5].

Generally, the stress response involves complex neuroendocrine pathways, primarily the hypothalamic-pituitary-adrenal (HPA) axis and the sympathetic-adrenal-medullary (SAM) axis. When stress activates the HPA axis, it triggers a cascade of hormonal responses. Corticotropin-releasing hormone (CRH) is released and ACTH is secreted from the anterior pituitary, which in turn prompts cortisol release from the adrenal glands [5,12,13]. Concurrently, stress activates the paraventricular nucleus of the hypothalamus, leading to the release of ADH from the posterior pituitary gland [12]. ADH acts synergistically to enhance CRH-induced ACTH secretion [12]. As reported in previous studies, this intricate interplay results in elevated serum levels of ACTH, ADH, and cortisol during vomiting attacks in CVS [13,14]. The possible factors that activated the HPA axis in this case were (1) physical stress due to tracheal intubation and surgical procedures, (2) direct stimulation of the HPA axis by adrenaline contained in local anesthetics, and (3) postoperative mental and physical stress.

In this case, serum ACTH, ADH, and cortisol levels remained unchanged after general anesthesia. The fact suggests that activation of the HPA axis due to tracheal intubation or surgical stress did not occur. The SAM axis, another key component of the stress response, releases catecholamines (epinephrine and norepinephrine) that can stimulate CRH release from the hypothalamus, thereby activating the HPA axis [15]. In this case, while we locally injected a small amount of adrenaline (a total of 67.5 µg of epinephrine) as an additive in local anesthetics, this exogenous adrenaline did not appear to affect HPA axis activation. This observation may contribute to the discussion regarding the threshold for the catecholamine-induced stress response in CVS patients. A characteristic of our perioperative management was the administration of midazolam to suppress postoperative agitation. Midazolam, a benzodiazepine, has been reported to suppress HPA axis activation via GABAergic neurons in the central nervous system, potentially reducing serum ACTH and cortisol levels in CVS patients [10,16,17]. Although midazolam was administered for a different purpose in our case, its HPA axis-inhibiting properties may have contributed to the suppression of postoperative vomiting. This serendipitous finding suggests that the strategic use of benzodiazepines could be a valuable tool in the perioperative management of CVS patients.

The patient's obesity presented an additional layer of complexity to this case. Obesity is known to increase the risk of vomiting through various mechanisms, including elevated intra-abdominal pressure, lower esophageal sphincter dysfunction, and increased gastric acid secretion [7-9]. Despite scheduling the surgery during a remission phase of vomiting attacks, in this case, the patient remained physically predisposed to vomiting due to these obesity-related factors. Therefore, a comprehensive approach to perioperative care that addresses both CVS and obesity-related risks was necessary. While our patient fortunately did not experience vomiting episodes during the perioperative period, this case emphasizes several areas for improvement. It has been recommended that triptans or serotonin antagonists be administered in the event of a vomiting attack caused by CVS [1,2]. The lack of prophylactic use of ondansetron or gastric antisecretory agents could be considered a limitation in this case. Another significant challenge in managing CVS patients perioperatively is determining the appropriate timing for surgery. Currently, we rely on the absence of characteristic CVS symptoms to identify non-seizure periods. There are no established objective criteria for defining non-seizure periods, making the determination largely based on patient reports and clinical judgment. This limitation underscores the need for more objective measures or biomarkers to guide surgical timing decisions in CVS patients.

## Conclusions

This case demonstrates successful management of general anesthesia in a patient with CVS and obesity, highlighting careful perioperative planning as follows: (1) adequate fasting, (2) scheduling surgery during non-seizure periods of CVS, (3) monitoring stress-related hormones, and (4) managing mental and physical stress. Moreover, the absence of significant changes in ACTH, ADH, and cortisol levels suggests that the anesthetic approach, including the use of midazolam, effectively suppresses HPA axis activation and prevents postoperative vomiting. While this case had a positive outcome, it suggests that prophylactic use of antiemetics or gastric antisecretory agents could be considered to further minimize risks.

## References

[1] Frazier R, Li BU, Venkatesan T (2023). Diagnosis and management of cyclic vomiting syndrome: a critical review. Am J Gastroenterol.

[2] Venkatesan T, Levinthal DJ, Tarbell SE (2019). Guidelines on management of cyclic vomiting syndrome in adults by the American Neurogastroenterology and Motility Society and the Cyclic Vomiting Syndrome Association. Neurogastroenterol Motil.

[3] Kovacic K, Li BU (2021). Cyclic vomiting syndrome: a narrative review and guide to management. Headache.

[4] Li BU, Balint JP (2000). Chapter 4 - Cyclic vomiting syndrome: evolution in our understanding of a brain-gut disorder. Adv Pediatr.

[5] Hejazi RA, McCallum RW (2011). Review article: cyclic vomiting syndrome in adults - rediscovering and redefining an old entity. Aliment Pharmacol Ther.

[6] Pareek N, Fleisher DR, Abell T (2007). Cyclic vomiting syndrome: what a gastroenterologist needs to know. Am J Gastroenterol.

[7] Khan A, Kim A, Sanossian C, Francois F (2016). Impact of obesity treatment on gastroesophageal reflux disease. World J Gastroenterol.

[8] Chang P, Friedenberg F (2014). Obesity and GERD. Gastroenterol Clin North Am.

[9] Nason KS (2015). Acute intraoperative pulmonary aspiration. Thorac Surg Clin.

[10] Garces J (2016). Anesthetic considerations for the patient with cyclic vomiting syndrome. AANA J.

[11] (2018). Headache Classification Committee of the International Headache Society (IHS). The International Classification of Headache Disorders, 3rd edition. Cephalalgia.

[12] Leistner C, Menke A (2020). Hypothalamic-pituitary-adrenal axis and stress. Handb Clin Neurol.

[13] Tarbell SE, Millar A, Laudenslager M, Palmer C, Fortunato JE (2017). Anxiety and physiological responses to the Trier Social Stress Test for Children in adolescents with cyclic vomiting syndrome. Auton Neurosci.

[14] Hikita T, Kodama H, Ogita K, Kaneko S, Nakamoto N, Mimaki M (2016). Cyclic vomiting syndrome in infants and children: a clinical follow-up study. Pediatr Neurol.

[15] Baritaki S, de Bree E, Chatzaki E, Pothoulakis C (2019). Chronic stress, inflammation, and colon cancer: a CRH system-driven molecular crosstalk. J Clin Med.

[16] Herman JP, McKlveen JM, Ghosal S (2016). Regulation of the hypothalamic-pituitary-adrenocortical stress response. Compr Physiol.

[17] Palmer GM, Cameron DJ (2005). Use of intravenous midazolam and clonidine in cyclical vomiting syndrome: a case report. Paediatr Anaesth.

